# Integration, scaling, space-group assignment and post-refinement

**DOI:** 10.1107/S0907444909047374

**Published:** 2010-01-22

**Authors:** Wolfgang Kabsch

**Affiliations:** a Max-Planck-Institut für Medizinische Forschung, Abteilung Biophysik, Jahnstrasse 29, 69120 Heidelberg, Germany

**Keywords:** *XDS*, integration, scaling, space-group assignment, post-refinement

## Abstract

Important steps in the processing of rotation data are described that are common to most software packages. These programs differ in the details and in the methods implemented to carry out the tasks. Here, the working principles underlying the data-reduction package *XDS* are explained, including the new features of automatic determination of spot size and reflecting range, recognition and assignment of crystal sym­metry and a highly efficient algorithm for the determination of correction/scaling factors.

## Abstract 2

The working principles of important steps in processing rotation data are described as employed by the program *XDS*.

## 1. Introduction   

The key steps in the processing of diffraction data from single crystals involve (i) modelling of the observed reflection positions in the detector plane, (ii) integration of diffraction intensities, (iii) data correction, scaling and post-refinement and (iv) space-group assignment. Much of the theory and many of the methods for carrying out these steps were developed about three decades ago for processing rotation data recorded on film and were subsequently extended in order to fully exploit the capabilities of a variety of electronic area detectors; some CCD (charge-coupled device) and multiwire detectors as well as a new pixel detector specially developed for data collection at synchrotron beamlines allow the recording of finely sliced rotation data because of their fast data read-out. In this article, the principles of the methods are described as employed by the program *XDS* (Kabsch, 2010 ▶). These apply equally well to rotation images covering small or large oscillation ranges. A large number of other data-processing systems have been developed which differ in the details of the implementations. Some of these packages were described in chapter 25.2 of Volume *F* of *International Tables for Crystallography* (2001 ▶). The theory and practice of pro­cessing fine-sliced data have been discussed by Pflugrath (1997 ▶).

## 2. Modelling rotation images   

The observed diffraction pattern, *i.e.* the positions of the reflections recorded on the rotation-data images, is controlled by a small set of parameters which must be accurately determined before integration can start. Approximate values for some of these parameters are given by the experimental setup, whereas others may be completely unknown and must be obtained from the rotation images. This is achieved by the automatic location of strong diffraction spots, the extraction of a primitive lattice basis that yields integer indices for the observed reflections and the subsequent refinement of all parameters to minimize the discrepancies between observed and calculated spot positions in the data images.

### 2.1. Coordinate systems and parameters   

In the rotation method, the incident-beam wavevector **S**_0_ of length 1/λ (where λ is the wavelength) is fixed while the crystal is rotated around a fixed axis described by a unit vector **m**_2_. **S**_0_ points from the X-ray source towards the crystal. It is assumed that the incident beam and the rotation axis intersect at one point at which the crystal must be located. This point is defined as the origin of a right-handed orthonormal laboratory co­ordinate system {**l**_1_, **l**_2_, **l**_3_}. This fixed but otherwise arbitrary system is used as a reference frame to specify the setup of the diffraction experiment.

Diffraction data are assumed to be recorded on a fixed planar detector. A right-handed orthonormal detector co­ordinate system {**d**_1_, **d**_2_, **d**_3_} is defined such that a point with coordinates *X*, *Y* in the detector plane is represented by the vector (*X* − *X*_0_)**d**_1_ + (*Y* − *Y*_0_)**d**_2_ + *F* **d**_3_ with respect to the laboratory coordinate system. The origin *X*_0_, *Y*_0_ of the detector plane is found at a distance |*F*| from the crystal position. It is assumed that the diffraction data are recorded on adjacent non-overlapping rotation images, each covering a constant oscillation range Δ_ϕ_, with image No. 1 starting at spindle angle ϕ_0_.

Diffraction geometry is conveniently expressed with respect to a right-handed orthonormal goniostat system {**m**_1_, **m**_2_, **m**_3_}. It is constructed from the rotation axis and the incident-beam direction such that **m**_1_ = (**m**_2_ × **S**_0_)/|**m**_2_ × **S**_0_| and **m**_3_ = **m**_1_ × **m**_2_. The origin of the goniostat system is defined to coincide with the origin of the laboratory system.

Finally, a right-handed crystal coordinate system {**b**_1_, **b**_2_, **b**_3_} and its reciprocal basis {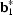, 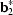, 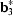} are defined to represent the unrotated crystal, *i.e.* at rotation angle ϕ = 0°, such that any reciprocal-lattice vector can be expressed as 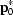 = 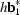 + 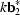 + 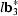, where *h*, *k*, *l* are integers.

As shown in §[Sec sec2.2]2.2, the location of all diffraction peaks recorded in the data images can be computed from the parameters **S**_0_, **m**_2_, **b**_1_, **b**_2_, **b**_3_, *X*_0_, *Y*_0_, *F*, **d**_1_, **d**_2_, **d**_3_, ϕ_0_ and Δ_ϕ_. In addition, knowledge of the shape and extent of the diffraction spots is required for accurate estimations of their intensities. This can be achieved by a Gaussian model involving two parameters: the standard deviations of the reflecting range, σ_M_, and of the beam divergence, σ_D_ (see §[Sec sec2.3]2.3). This leads to an integration region around the spot defined by the parameters δ_M_ and δ_D_, which are typically chosen to be 6–10 times larger than σ_M_ and σ_D_, respectively.

### 2.2. Spot prediction   

Let 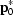 denote any arbitrary reciprocal-lattice vector if the crystal has not been rotated, *i.e.* at rotation angle ϕ = 0°. Depending on the diffraction geometry, 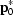 may be rotated into a position fulfilling the reflecting condition. The required rotation angle ϕ and the coordinates *X*, *Y* of the diffracted beam at its intersection with the detector plane can be found from 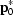 as follows.


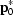
 can be expressed by its components with respect to the orthonormal goniostat system as 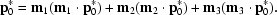
Rotation by ϕ around axis **m**_2_ changes 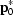 into 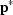, 
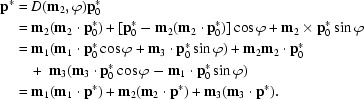
The incident-beam and diffracted-beam wavevectors, **S**_0_ and **S**, have their termini on the Ewald sphere and satisfy the Laue equations 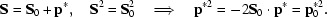
If ρ = 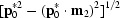 denotes the distance of 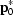 from the rotation axis, solutions for 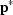 and ϕ can be obtained in terms of 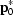 as 
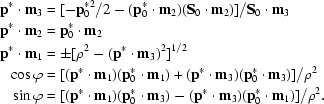
In general there are two solutions according to the sign of 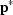
·**m**_1_. If ρ^2^ < (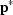
·**m**_3_)^2^ or 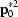 > 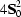 the Laue equations have no solution and the reciprocal-lattice point 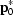 is in the ‘blind’ region.

If *F* **S**·**d**_3_ > 0 the diffracted beam intersects the detector plane at the point 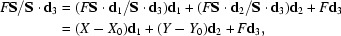
which leads to a diffraction spot recorded at detector co­ordinates 
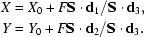



### 2.3. Standard spot shape   

A reciprocal-lattice point crosses the Ewald sphere by the shortest route only if the crystal happens to be rotated about an axis perpendicular to both the diffracted-beam and incident-beam wavevectors, the ‘β axis’ **e**_1_ = **S** × **S**_0_/|**S** × **S**_0_|, as introduced by Schutt & Winkler (1977 ▶). Rotation around the fixed axis **m**_2_, as enforced by the rotation camera, thus leads to an increase in the length of the shortest path by the factor 1/|**e**_1_·**m**_2_|. This motivated the introduction of a coordinate system {**e**_1_, **e**_2_, **e**_3_}, specific for each reflection, which has its origin on the surface of the Ewald sphere at the terminus of the diffracted beam wavevector **S**, 
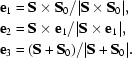
The unit vectors **e**_1_ and **e**_2_ are tangential to the Ewald sphere, while **e**_3_ is perpendicular to **e**_1_ and 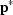 = **S** − **S**_0_. The shape of a reflection, as represented with respect to {**e**_1_, **e**_2_, **e**_3_}, then no longer contains geometrical distortions resulting from the fixed rotation axis of the camera and the oblique incidence of the diffracted beam on a flat detector. Instead, all reflections appear as if they had followed the shortest path through the Ewald sphere and had been recorded on the surface of the sphere.

A detector pixel at *X*′, *Y*′ in the neighbourhood of the reflection centre *X*, *Y*, when the crystal is rotated by ϕ′ instead of ϕ, is mapped to the profile coordinates ∊_1_, ∊_2_, ∊_3_ by the following procedure: 
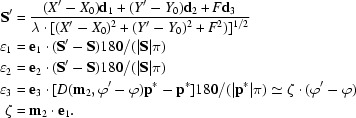
ζ corrects for the increased path length of the reflection through the Ewald sphere and is closely related to the reciprocal Lorentz correction factor 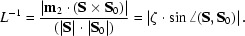
Because of crystal mosaicity and beam divergence, the intensity of a reflection is smeared around the diffraction maximum. The fraction of total reflection intensity found in the volume element d∊_1_d∊_2_d∊_3_ at ∊_1_, ∊_2_, ∊_3_ can be approximated by Gaussian functions: 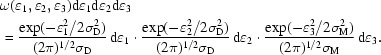



### 2.4. Spot centroids and partiality   

The intensity of a reflection can be completely recorded on one image or distributed among several adjacent images. The fraction *R_j_* of total intensity recorded on image *j*, the ‘partiality’ of the reflection, can be derived from the distribution function ω(∊_1_, ∊_2_, ∊_3_) as 
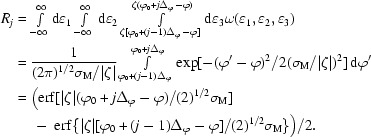
The integral is evaluated by using a numerical approximation of the error function, erf (Abramowitz & Stegun, 1972 ▶).

While the spot centroids in the detector plane are usually good estimates for the detector position of the diffraction maximum, the angular centroid about the rotation axis, 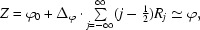
can be a rather poor guess for the true ϕ angle of the maximum. Its accuracy depends strongly on the value of ϕ and the size of the oscillation range Δϕ relative to the mosaicity σ_M_ of the crystal. For a reflection fully recorded on image *j*, the value *Z* = ϕ_0_ + (*j* − ½)·Δ_ϕ_ will always be obtained, which is correct only if ϕ accidentally happens to be close to the centre of the rotation range of the image. In contrast, the ϕ angle of a partial reflection recorded on images *j* and *j* + 1 is closely approximated by *Z* = ϕ_0_ + [*j* + (*R*_*j*+1_ − *R_j_*)/2]·Δ_ϕ_. If many images contribute to the spot intensity, *Z*(ϕ) is always an excellent approximation to the ideal angular position ϕ when the Laue equations are satisfied; in fact, in the limiting case of infinitely fine-sliced data it can be shown that lim_Δϕ→0_ *Z*(ϕ) = ϕ.

Most refinement routines minimize the discrepancies between the predicted ϕ angles and their approximations obtained from the observed *Z* centroids and must therefore carefully distinguish between fully and partially recorded reflections. However, this distinction is unnecessary if the observed *Z* centroids are instead compared with their analytic forms, because the sensitivity of the centroid positions to the diffraction parameters is correctly weighted in either case (see §[Sec sec2.8]2.8).

### 2.5. Localizing diffraction spots   

Often, some of the parameters controlling the diffraction experiment are either completely unknown or available only at a crude approximation. Accurate values for all parameters must be obtained from the recorded data, *i.e.* from a list of the coordinates of strong spots occurring in the images. As implemented in *XDS*, this list is obtained from all or a subset of the data images by the following procedure. Firstly, each pixel value is compared with the mean value and standard deviation of surrounding pixels in the same image and classified as a strong pixel if its value exceeds the mean by a given multiple (typically 3–5) of the standard deviation. Values of the strong pixels and their location addresses and image running numbers are saved in a file. After the scan, a hash table of sufficient size is allocated to accommodate the strong pixels from the file together with their addresses (for a dis­cussion of the hash technique, see Wirth, 1976 ▶). As several strong pixels may belong to the same spot, they are labelled with a unique spot number so that any two such pixels which can be connected by direct strong neighbours in two or three dimensions (if there are adjacent images) belong to the same spot (equivalence class). The labelling is achieved by the highly efficient algorithm for the recording of equivalence classes developed by Rem (see Dijkstra, 1976 ▶). On termination, a list *X*′*_i_*, *Y*′*_i_*, *Z*′*_i_* (*i* = 1, …, *n*) of the centroids of *n* strong spots is available.

### 2.6. Basis extraction   

Any reciprocal-lattice vector can be written in the form 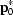 = 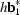 + 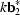 + 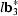, where *h*, *k*, *l* are integers and 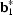, 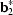, 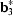 are reciprocal basis vectors of the lattice. The basis vectors which describe the orientation, metric and symmetry of the crystal, as well as the reflection indices *h*, *k*, *l*, have to be determined from the list of strong diffraction spots *X*′*_i_*, *Y*′*_i_, Z*′*_i_* (*i* = 1, …, *n*). Ideally, each spot corresponds to a reciprocal-lattice vector 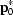 which satisfies the Laue equations after a crystal rotation by ϕ. Substituting the observed value *Z*′ for the unknown ϕ angle (see §[Sec sec2.4]2.4), 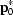 is found from the observed spot coordinates as 
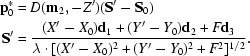
Unfortunately, the reciprocal-lattice vectors 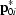 (*i* = 1, …, *n*) derived from the above list of strong diffraction spots often contain a number of ‘aliens’ (spots arising from fluctuations in the background, from ice or from satellite crystals) and a robust method has to be used which is still capable of recognizing the dominant lattice. One approach, suggested by Bricogne (1986 ▶) and implemented in a number of variants (Otwinowski & Minor, 1997 ▶; Steller *et al.*, 1997 ▶), is to identify a lattice basis as the three shortest linear independent vectors **b**_1_, **b**_2_, **b**_3_, each at a maximum of the Fourier transform 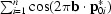. Alternatively, a reciprocal basis for the dominant lattice can be determined from short differences between the reciprocal-lattice vectors (Howard, 1986 ▶; Kabsch, 1988*a* ▶). As implemented in *XDS*, a lattice basis is found by the following procedure.

The list of given reciprocal-lattice points 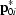 (*i* = 1, …, *n*) is first reduced to a small number *m* of low-resolution difference-vector clusters 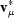 (μ = 1, …, *m*). *f*_μ_ is the population of a difference-vector cluster 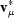; that is, the number of times the difference between any two reciprocal-lattice vectors 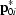 − 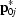 is approximately equal to 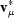. In a second step, three linear independent vectors 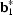, 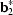, 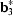 are selected among all possible triplets of difference-vector clusters that maximize the function *Q*, 
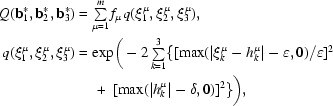
where
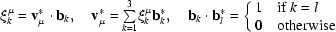
and *h_k_* ^μ^ is the nearest integer to ξ*_k_* ^μ^. The absolute maximum of *Q* is assumed if all difference vectors can be expressed as small integral multiples of the best triplet. Deviations from this ideal situation are quantified by the quality measure *q*. The value of *q* declines sharply if the expansion coefficients ξ*_k_* ^μ^ deviate by more than ∊ from their nearest integers *h_k_* ^μ^ or if the indices are absolutely larger than δ. The constraint on the allowed range of indices prevents the selection of a spurious triplet of very short difference-vector clusters which might be present in the set. Excellent results have been obtained using ∊ = 0.05 and δ = 5. The best vector triplet thus found is refined against the observed difference-vector clusters. Finally, a reduced cell is derived from the refined reciprocal-base vector triplet (see §[Sec sec6]6).

### 2.7. Indexing   

Once a basis **b**_1_, **b**_2_, **b**_3_ of the lattice is available, integral indices *h_i_*, *k_i_*, *l_i_* must be assigned to each reciprocal-lattice vector 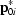 (*i* = 1, …, *n*). Using the integers nearest to 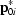
·**b** *_k_* (*k* = 1, 2, 3) as indices of the reciprocal-lattice vectors 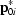 could easily lead to a misindexing of longer vectors because of inaccuracies in the basis vectors **b** *_k_* and the initial values of the parameters describing the instrumental setup. A more robust solution of the indexing problem is provided by the local indexing method, which assigns only small index differences *h_i_* − *h_j_*, *k_i_* − *k_j_*, *l_i_* − *l_j_* between pairs of neighbouring reciprocal-lattice vectors (Kabsch, 1993 ▶).

The reciprocal-lattice points can be considered as nodes of a tree. The tree connects the *n* points to each other with the connections as its branches. The length ℓ*_ij_* of a possible branch between nodes *i* and *j* is defined here as 
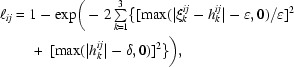
where 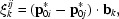

*h_k_^ij^* is the nearest integer of ξ*_k_^ij^* and *k* = 1, 2, 3. Reliable index differences are indicated by short branches; in fact, ℓ*_ij_* is 0 if none of the indices *h_k_^ij^* is absolutely larger than δ and the ξ*_k_^ij^* are integer values to within ∊. Typical values are ∊ = 0.05 and δ = 5. Defining the length of a tree as the sum of the lengths of its branches, a shortest tree among all *n* ^*n*−2^ possible trees is determined using the elegant algorithm described by Dijkstra (1976 ▶). Starting with arbitrary indices 0, 0, 0 for the root node, the local indexing method then consists of traversing the shortest tree and thereby assigning each node the indices of its predecessor plus the small index differences between the two nodes.

During traversal of the tree, each node is also given a subtree number. Starting with subtree number 1 for the root node, each successor node is given the same subtree number as its predecessor if the length of the connecting branch is below a minimal length ℓ_min_. Otherwise, its subtree number is incremented by 1. Thus, all nodes in the same subtree have internally consistent reflection indices. Defining the size of a subtree by the number of its nodes, ‘aliens’ are usually found in small subtrees. Finally, a constant index offset is determined such that the centroids of the observed reciprocal-lattice points 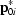 belonging to the largest subtree and their corresponding grid vectors 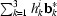 are as close as possible. This offset is added to the indices of each reciprocal-lattice point.

### 2.8. Refinement   

For a fixed detector, the diffraction pattern depends on the parameters **S**_0_, **m**_2_, **b**_1_, **b**_2_, **b**_3_, *X*_0_, *Y*_0_, *F*. Starting values for the parameters can be obtained by the procedures described above, which do not rely on prior knowledge of the crystal orientation, space-group symmetry or unit-cell metric. Better estimates of the parameter values, as required for the subsequent integration step, can be obtained by the method of least squares from the list of *n* observed indexed reflection centroids *h_i_, k_i_, l_i_, X_i_*′, *Y_i_*′, *Z_i_*′ (*i* = 1, …, *n*). In this method, the parameters are chosen to minimize a weighted sum of squares of the residuals 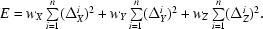
The residuals between the calculated (*X_i_, Y_i_, Z_i_*) and observed spot centroids are 
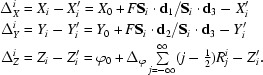
Let *s*_μ_ (μ = 1, …, *k*) denote the *k* independent parameters for which initial estimates are available. Expanding the residuals to first order in the parameter changes 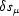 gives 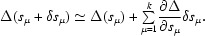
The parameters should be changed in such a way as to minimize 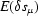, which implies 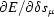 = 0 for μ = 1, …, *k*. The 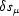 are found as the solution of the *k* normal equations 
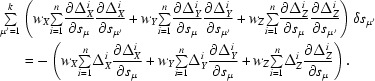
The parameters are corrected by 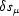 and a new cycle of refinement is started until a minimum of *E* is reached. The weights 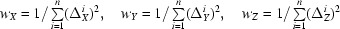
are calculated with the current guess for *s*_μ_ at the beginning of each cycle.

The derivatives appearing in the normal equations can be worked out from the definitions given in §§[Sec sec2.2]2.2 and [Sec sec2.4]2.4 and only the form of the gradient of the *Z* residuals is shown. Assuming σ*_i_* = σ_M_/|ζ*_i_*| (*i* = 1, …, *n*) is constant for each reflection, the gradients of the *Z* residuals are obtained from the chain rule and the relation derf(*z*)/d*z* = (2/π^1/2^)exp(−*z* ^2^). 
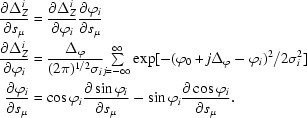
Obviously, ∂Δ*^i^_Z_*/∂*s*_μ_ is small for a fully recorded reflection because of the small values of all exponentials appearing in ∂Δ*^i^_Z_*/∂ϕ_*i*_. In contrast, the gradient for a partial reflection that is equally recorded on two adjacent images is most sensitive to parameter variations because one of the exponentials assumes its maximum value. In the limiting case of infinitely fine-sliced data it can be shown that lim_Δϕ→0_∂Δ*^i^_Z_*/∂ϕ__i__ = 1. Thus, the refinement scheme based on observed *Z* centroids, as described here and implemented in *XDS*, is applicable to fine-sliced data and also to data recorded with a large oscillation range.

## 3. Integration   

Assuming that the diffraction parameters have been refined successfully as described above, the intensity of a reflection is distributed in the neighbourhood of the predicted location of the diffraction peak among detector pixels of one or several adjacent rotation images. Accurate integration requires several steps: determination of a reflection mask, estimation of the background, generation of reference profiles and integration by profile fitting.

The intensity distribution of a reflection can be modelled analytically or derived from the observed profiles of neighbouring strong spots. For the rotation method, the profile shape depends strongly on the specific path of the reflection through the Ewald sphere and on variations in the angle of incidence of the diffracted beam on a flat detector. These geometrical distortions can be eliminated by mapping the reflections onto the coordinate system defined in §[Sec sec2.3]2.3, which simplifies the task of modelling the expected intensity distribution as all reflection profiles become similar.

### 3.1. Reflection mask   

The parameters σ_M_ and σ_D_ of the Gaussian model (see §[Sec sec2.3]2.3) used to describe reflection shape can be determined automatically from one or more data images by the following procedure.

(i) Identify and mark strong pixels in the data image.
(ii) Assign the indices of the nearest reflection to each strong pixel.
(iii) Sort the strong pixels by the assigned reflection indices such that pixels with the same indices follow each other in the list.
(iv) For each strong reflection find the rectangular box that encloses all of the strong pixels belonging to the reflection.
(v) Increase the box slightly and use all pixels within the box that are not strong for background determination.
(vi) Subtract the background and determine the centroid and variance *s* ^2^ of the intensity-weighted diffracted-beam directions λ**S**′ associated with each strong pixel belonging to the spot (see §[Sec sec2.3]2.3).
(vii) Reject the spot if the centroid position deviates too much from the calculated spot location.
(viii) Calculate ϕ and ζ for the accepted reflection and save the three values ϕ, ζ and *s* ^2^ in a list.

The standard deviation of the beam divergence is obtained directly from this list of *n* reflections as 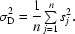
Determination of the standard deviation of the reflecting range, the mosaicity σ_M_, requires additional considerations. For each of the *n* reflections from the list above, let τ denote the angular difference between the rotation angle ϕ at its Bragg maximum and the centre of the oscillation angles covered by the image. The fraction of observed reflection intensity is (see §[Sec sec2.4]2.4) 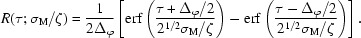
For a given σ_M_/ζ the function *R*(τ; σ_M_/ζ) assumes its maximum at τ = 0 and declines as |τ| increases. The decline depends strongly on the mosaicity σ_M_ and on the path length of the reflection through the Ewald sphere, which is accounted for by the factor 1/ζ. For a large mosaicity *R*(τ; σ_M_/ζ) declines slowly, which explains why for such crystals many reflections with large |τ| values can be observed on a data image. Clearly, the list of strong spots located by the automatic procedure described above contains information about the mosaicity of the crystal. The problem of finding σ_M_ from this list can be solved if one considers each τ value as a random variable drawn from a probability distribution *R*(τ; σ_M_/ζ) with population parameter σ_M_/ζ. The mosaicity σ_M_ can then be estimated so that it maximizes the likelihood (joint probability) 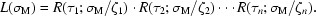
The parameters σ_D_ and σ_M_ are mainly used to specify the integration region around the spot defined by the parameters δ_M_ and δ_D_, which are typically chosen to be 6–10 times larger than σ_M_ and σ_D_, respectively (see §[Sec sec2.1]2.1). The reflection mask thus comprises all image pixels that satisfy 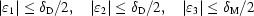
when mapped to the profile coordinate system {**e**_1_, **e**_2_, **e**_3_} defined in §[Sec sec2.3]2.3. In addition, pixels are excluded from the mask if they are closer to the predicted Bragg peak of an intruding reflection from the neighbourhood.

### 3.2. Background   

The region around a spot is assumed to have been chosen to be large enough to include a sufficient number of pixels which can be used for determination of the background. Background determination, as implemented in *XDS*, begins by sorting all pixels belonging to a reflection by increasing intensity. For weak or absent reflections, these values should represent a random sample drawn from a normal distribution. If this is not the case, the pixel with the largest intensity is removed until the sampling distribution of the remaining smaller items satisfies the expected distribution. This method will also exclude pixels with unexpected high values, such as ice reflections. The background, determined as the mean value of the accepted pixels, is systematically overestimated for strong spots because of some residual intensity extending into the accepted background pixels. This residual intensity is estimated from the expected distribution ω(∊_1_, ∊_2_, ∊_3_) defined in §[Sec sec2.3]2.3 and removed from the final background value.

### 3.3. Standard profiles   

Reflection profiles are represented on the Ewald sphere within a domain *D*_0_ comprising 2*n*_1_ + 1, 2*n*_2_ + 1 and 2*n*_3_ + 1 equidistant gridpoints along **e**_1_, **e**_2_ and **e**_3_, respectively. The sampling distances between adjacent grid points are then Δ_1_ = δ_D_/(2*n*_1_ + 1), Δ_2_ = δ_D_/(2*n*_2_ + 1) and Δ_3_ = δ_M_/(2*n*_3_ + 1). Thus, grid coordinate ν_3_ (ν_3_ = −*n*_3_, …, *n*_3_) covers the set of rotation angles 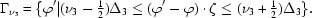
Contributions to the spot intensity come from one or several adjacent data images (*j* = *j*_1_, …, *j*_2_), each covering the set of rotation angles 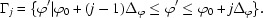
Assuming Gaussian profiles along **e**_3_ for all reflections (see §[Sec sec2.3]2.3), the fraction of counts (after subtraction of the background) contributed by data frame *j* to grid coordinate ν_3_ is 
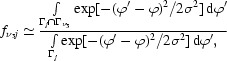
where σ = σ_M_/|ζ|. The integrals can be expressed in terms of the error function, for which efficient numerical approximations are available (Abramowitz & Stegun, 1972 ▶). Finally, each pixel in data image *j* belonging to the reflection is subdivided into 5 × 5 areas of equal size and 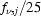 of the pixel signal is added to the profile value at grid coordinates ν_1_, ν_3_, ν_3_ corresponding to each subdivision.

This complicated procedure leads to more uniform intensity profiles for all reflections than using their untransformed shape. This simplifies the task of modelling the expected intensity distribution needed for integration by profile fitting. As implemented in *XDS*, reference profiles are learnt every 5° of crystal rotation at nine positions on the detector, each covering an equal area of the detector face. In the learning phase, profile boxes of the strong reflections are normalized and added to their nearest reference profile boxes. The con­tributions are weighted according to the distance from the location of the reference profile. Each grid point within the average profile boxes is classified as signal if it is above 2% of the peak maximum. Finally, each profile is scaled such that the sum of its signal pixels normalizes to one. The analytical expression ω(∊_1_, ∊_2_, ∊_3_) defined in §[Sec sec2.3]2.3 for the expected intensity distribution is only a rough initial approximation, which is now replaced by the empirical reference profiles.

### 3.4. Intensity estimation   

If an expected intensity distribution {*p_i_*|*i* ∈ *D*_0_} of the observed profile is given in a domain *D*_0_, the reflection intensity *I* can be estimated as 
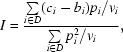
which minimizes the function 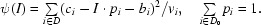

*b*_*i*_, *c*_*i*_, *v*_*i*_ (*i* ∈ *D*) are the background, contents and variance of pixels observed in a subdomain *D* 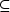 *D*_0_ of the expected distribution. The background *b_i_* underneath a diffraction spot is often assumed to be a constant which is estimated from the neighbourhood around the reflection. Determination of reflection intensities by profile fitting has a long tradition (Diamond, 1969 ▶; Ford, 1974 ▶; Kabsch, 1988*b* ▶; Otwinowski, 1993 ▶). Implementations of the method differ mainly in their assumptions about the variances *v_i_*. Ford used constant variances, which work well for films, which have a high intrinsic background. In *XDS*, which was originally designed for a multwire detector, *v_i_* ∝ *p_i_* was assumed, which results in a straight summation of background-subtracted counts within the expected profile region, *I* = 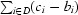/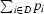. This particular simple formula is very satisfactory for the low background typical of these detectors. For the general case, however, better results can be obtained by using *v_i_* = *b_i_* + *Ip_i_* for the pixel variances as shown by Otwinowski and implemented in *DENZO* and in later versions of *XDS*. Starting with *v_i_* = *b_i_*, the intensity is now found by an iterative process which is terminated if the new intensity estimate becomes negative or does not change within a small tolerance, which is usually reached after three cycles. It can be shown that the solution thus obtained is unique.

## 4. Scaling   

The integrated intensities of the reflections need to be corrected by various factors arising from the following

(i) changes in the intensity of the incident beam and variations in the illuminated crystal volume,
(ii) absorption of incident and diffracted beams,
(iii) radiation damage,
(iv) variations in detector sensitivity within the detector plane and
(v) different crystal sizes and crystalline order if the data are from several crystals.

The combined effect manifests itself in correlations of the intensity of a reflection with details of its measurement, such as time (or image number) and location in the detector plane. Usually, many statistically independent observations of symmetry-related reflections are recorded in the rotation images taken from one or several similar crystals of the same compound. The squared structure-factor amplitudes of equivalent reflections should be equal and many scaling procedures (see, for example Evans, 2006 ▶; Otwinowski *et al.*, 2003 ▶; Kabsch, 1988*b* ▶) exploit this *a priori* knowledge to determine a correction factor for each observed intensity. However, the scaling programs differ in the details of their scaling models, *i.e.* the parametrization and methods used for determination of the correction factors. Below, the approach is described as implemented recently in the programs *XDS* and *XSCALE* (Kabsch, 2010 ▶).

If more than one data set is included, these are first put on approximately the same scale by the factor *K*·exp[*B*·(2sinθ/λ^2^)] involving two parameters, *K* and *B*, for each data set. The parameter values are assigned so that the resulting correction factors fit best to the observed intensity ratios of common reflections in each pair of data sets.

For the more detailed corrections, three types of two-dimensional functions are used in succession to remove correlations of the intensity of a reflection with (i) image number and resolution, (ii) location in the detector plane and (iii) image number and 13 detector surface regions. To correct for non-uniform detector response such as edge effects at the boundaries of multisegment detectors, the use of smooth analytical correction functions was avoided. Instead, the correction functions are sampled at a finite set of grid regions covering all of the function’s definition range. The grid regions are chosen automatically to be as small as possible without overfitting the data so that each sampling region contains more than a specified minimum number of reflections (default 50). Thus, the correction function *G* is represented by a possibly large number of reciprocal factors *G_l_*, where the subscript *l* denotes the grid regions.

The correction factors *G_l_* are found in a cyclic prodedure starting with *G_l_* = 1. In each cycle, *G_l_* is updated by a factor *g_l_*. The target function for refinement is based on an observational equation for each reflection 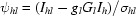
as introduced by Hamilton *et al.* (1965 ▶). The subscript *h* represents the unique reflection indices and *hl* denotes symmetry-related reflections to *h* that need to be corrected by the reciprocal scaling factor *g_l_* associated with grid point *l*; *I_hl_* and σ*_hl_* are their weighted mean and standard deviation, respectively. This standard deviation is considered to be infinitely large if no such reflection was measured, which amounts to omitting the observational equation altogether. The factors 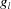 and the ‘true’ intensities 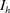 are found at the minimum of the function 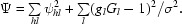
The first sum on the right side is a homogeneous function of *g_l_* of degree zero so that the *g_l_* would only be determined up to an arbitrary factor. The second sum on the right side is used to weakly restrain the scaling factors to one; a reasonable value is σ = 0.05. Minimization of Ψ leads to updates *g_l_* in terms of the ‘true’ intensities *I_h_* which again depend on *g_l_*, 
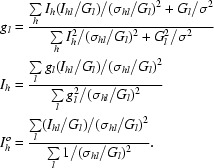
The new update factors *g_l_* are obtained by using ‘true’ intensities *I_h_^o^* from the previous cycle instead of the current *I_h_* as defined above. At the end of the cycle, the old correction factors *G_l_* are updated by multiplication with the new *g_l_*. This cyclic procedure typically converges in less than six cycles.

The approach described here has been implemented in *XDS* and *XSCALE* and has been successfully used for more than two years. In contrast to the ‘shortest path’ eigenvector method of Fox & Holmes (1966 ▶), which is very efficient for a relatively small number of variables, the computations here require a time that is proportional to the number of reflections used for scaling and thus quickly lead to a solution even when a very large number of correction factors from many data sets are involved.

## 5. Post-refinement   

The number of fully recorded reflections on each single image rapidly declines for small oscillation ranges and the complete intensities of the partially recorded reflections have to be estimated. This presented a serious obstacle in early structural work on virus crystals, as the crystal had to be replaced after each exposure on account of radiation damage. A solution to this problem, the ‘post-refinement’ technique, was found by Schutt, Winkler and Harrison and variants of this powerful method have been incorporated into most data-reduction programs (for a detailed discussion, see Harrison *et al.*, 1985 ▶; Rossmann, 1985 ▶). The method derives complete intensities of reflections that are only partially recorded on an image from accurate estimates for the fractions of observed intensity: the ‘partiality’. The partiality of each reflection can always be calculated as a function of orientation, unit-cell metric, mosaic spread of the crystal and model intensity distributions. The accuracy of the estimated full reflection intensity obviously then strongly depends on a precise knowledge of the parameters describing the diffraction experiment. Usually, symmetry-related fully recorded reflections can be found for many of the partial reflections and the list of such pairs of intensity observations can be used to refine the required parameters using a least-squares procedure. Clearly, this refinement is carried out after all images have been processed, which explains why the procedure is called ‘post-refinement’.

Adjustments of the diffraction parameters *s*_μ_ (μ = 1, …, *k*) are determined by minimization of the function *E*, which is defined as the weighted sum of squared residuals between calculated and observed partial intensites. 
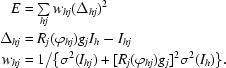
Here, *I_hj_* is the intensity recorded on image *j* of a partial reflection with indices summarized as *hj*, *I_h_* is the mean of the observed intensities of all fully recorded reflections symmetry-equivalent to *hj*, *g*_*j*_ is the inverse scaling factor of image *j*, ϕ_*hj*_ is the calculated spindle angle of reflection *hj* at diffraction and *R_j_* is the computed fraction of total intensity recorded on image *j*.

Expansion of the residuals Δ*_hj_* to first order in the parameter changes δ*s*_μ_ and minimization of *E*(δ*s*_μ_) leads to the *k* normal equations 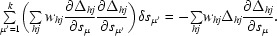
Often, the normal matrix is ill-conditioned since changes in some unit-cell parameters or small rotations of the crystal about the incident X-ray beam do not significantly affect the calculated partiality *R_j_*. To take care of these difficulties, the system of equations is rescaled to yield unit diagonal elements for the normal matrix and the correction vector δ*s*_μ_ is filtered by projection into a subspace defined by the eigenvectors of the normal matrix with sufficiently large eigenvalues (Diamond, 1966 ▶).

The parameters are corrected by the filtered δ*s*_μ_ and a new cycle of refinement is started until a minimum of *E* is reached. The weights, residuals and their gradients are calculated using the current values for *s*_μ_ and *g_j_* at the beginning of each cycle. The derivatives 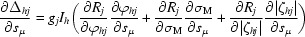
appearing in the normal equations can be worked out from the definitions given in §§[Sec sec2.2]2.2 and [Sec sec2.4]2.4 (to simplify the following equations, the subscript *hj* is omitted). The fraction *R_j_* of total intensity can be expressed in terms of the error function (see §[Sec sec2.4]2.4) as 
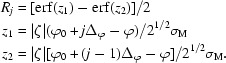
Using the relation derf(*z*)/d*z* = (2/π^1/2^)exp(−*z* ^2^), the derivatives of *R_j_* are 
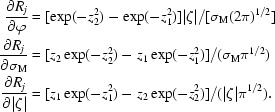
The derivatives ∂ϕ/∂*s*_μ_, ∂σ_M_/∂*s*_μ_ and ∂|ζ|/∂*s*_μ_ remain to be worked out (not shown here). As discussed in detail by Greenhough & Helliwell (1982 ▶), spectral dispersion and asymmetric beam cross-fire lead to some variation in σ_M_, which makes it necessary to include additional parameters in the list *s*_μ_. The effect of these parameters on the partiality is dealt with easily by the derivatives ∂σ_M_/∂*s*_μ_.

The refinement scheme described above requires initial scaling factors *g_j_*. With the now improved estimates for the partialities *R_j_*, a new set of scaling factors can be obtained using the method outlined in §[Sec sec4]4. This alternating procedure of scaling and post-refinement usually converges within three cycles.

The use of error functions for modelling partiality, as implicated by a Gaussian model for describing spot shape, was chosen here for reasons of conceptual simplicity and coherence. This choice is unlikely to significantly alter the results of post-refinement that are based on other functions of similar form (see the discussion by Rossmann, 1985 ▶).

## 6. Space-group assignment   

Identification of the correct space group is not always an easy task and should be postponed for as long as possible. Sometimes, the true space group only becomes known when the structure has been successfully solved and refined! However, one can expect to identify a small number of possibilities from the diffraction experiment.

Fortunately, all data processing as implemented in the program *XDS* can be carried out in the absence of any knowledge of the crystal symmetry and unit-cell parameters. In this case, a reduced cell is extracted from the observed diffraction pattern and processing of the data images con­tinues to completion as if the crystal were triclinic. Clearly, the reflection indices then refer to the reduced cell and must be re­indexed once the space group is known. For all space groups, the required reindexing transformation is linear and involves only whole numbers, as shown in Part 9 of Vol. *A* of *International Tables for Crystallography* (1989 ▶).

Automatic space-group assignment is carried out in two steps once integrated intensities of all reflections are available (see Kabsch, 2010 ▶). Firstly, the Bravais lattices are identified that are compatible with the reduced cell derived from the observed diffraction pattern. In the second step, all enantiomorphous space groups compatible with the observed lattice symmetry are rated by a redundancy-independent *R* factor. The group is selected that explains all integrated intensities in the data set at an acceptable *R* factor requiring a minimum number of unique reflections (Occam’s principle). This approach deliberately avoids any test for the presence of screw axes as these tests would depend strongly on the com­pleteness of the data. Fortunately, the presence or absence of screw axes is irrelevant for the determination of data correction/scaling factors (see §[Sec sec4]4).

### 6.1. Determination of the Bravais lattice   

The determination of possible Bravais lattices is based upon the concept of the reduced cell whose metric parameters characterize 44 lattice types as described in Part 9 of Vol. *A* of *International Tables for Crystallography* (1989 ▶). A primitive basis **b**_1_, **b**_2_, **b**_3_ of a given lattice is defined there as a reduced cell if it is right-handed and if the components of its metric tensor 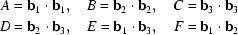
satisfy a number of conditions (inequalities). The main con­ditions state that the basis vectors are the shortest three linear independent lattice vectors with either all acute or all non-acute angles between them. As specified in *International Tables for Crystallography*, each of the 44 lattice types is characterized by additional equality relations among the six components of the reduced-cell metric tensor. As an example, for lattice character 11 (Bravais type *tP*) the components of the metric tensor of the reduced cell must satisfy 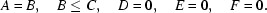
(Note that the other tetragonal primitive lattice character 21 requires *A* ≤ *B* = *C* with the fourfold as the shortest axis.)

Any primitive triclinic cell describing a given lattice can be converted into a reduced cell. It is well known, however, that the reduced cell thus derived is sensitive to experimental error. Hence, the direct approach of first deriving the correct reduced cell and then finding the lattice type is unstable and may in certain cases even prevent identification of the correct Bravais lattice.

A suitable solution of the problem has been found that avoids any decision as to what the ‘true’ reduced cell is (see Kabsch, 1993 ▶). The essential ingredients of this procedure are (i) a database of possible reduced cells and (ii) a backward-search strategy that finds the best-fitting cell in the database for each lattice type.

The database is derived from a seed cell which strictly satisfies the definitions for a reduced cell. All cells of the same volume as the seed cell whose basis vectors can be linearly expressed in terms of the seed vectors by indices −1, 0 or +1 are included in the database. Each unit cell in the database is considered as a potential reduced cell, although some of the defining conditions as given in Part 9 of Vol. *A* of *International Tables for Crystallography* (1989 ▶) may be violated. These violations are treated as arising from experimental error.

The backward-search strategy starts with the hypothesis that the lattice type is already known and identifies the best-fitting cell in the database of possible reduced cells. In contrast to a forward-directed search, it is now always possible to decide which conditions have to be satisfied by the components of the metric tensor of the reduced cell. The total amount by which all these equality and inequality conditions are violated is used as a quality index. For example, to find out how well a potential reduced cell **b**_1_, **b**_2_, **b**_3_ from the database characterizes lattice character 11 (Bravais lattice *tP*), the quality index 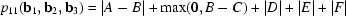
is computed. Positive values of *p*_11_ indicate that some conditions are not satisfied. All potential reduced cells in the data base are tested and the smallest value for *p*_11_ is used for rating lattice type 11. A similar test is carried out for all 44 possible lattice types using quality indices based on their defining conditions as listed in Part 9 of Vol. *A* of *International Tables for Crystallography* (1989 ▶).

The results obtained using this method are shown in Table 1 ▶ for the example of a data set comprising 177 images with each exposure covering 0.5° of spindle rotation. The space group of the protein crystal was *P*4_3_2_1_2 (unit-cell parameters *a* = 159.4, *b* = 159.4, *c* = 160.3 Å), but this knowledge was not used in the processing. Instead, the data were processed with respect to a triclinic reduced cell derived from the observed diffraction pattern as described above. The images contained a total of 292 998 reflections within the resolution range 20.0–3.0 Å; 57 548 reflections in the resolution range 10.0–5.0 Å were used for space-group determination. For determination of the lattice symmetry all 44 possibilities were considered and rated by their quality index. The table shows the possible lattice sym­metries, their implied conventional unit-cell parameters and a reindexing transformation. The table entries are sorted by increasing quality index and reveal a nearly cubic lattice symmetry. A lattice symmetry is considered to be acceptable if it has a low quality index and its implied unit-cell parameters do not violate the ideal values by more than 3.0° in angles and 3% in cell axes. Thus, except for the last entry, all of the lattice symmetries in the table are acceptable; the correct lattice type 11 *tP* is highlighted. Lattice symmetries that are not accepted include all body-centred lattices or those that are centred on all faces; they are omitted from the table.

The reindexing transformation REIDX() consists of 12 integers that relate the original indices *h*, *k*, *l* used during the integration to the indices *h*′, *k*′, *l*′ with respect to the new cell. 
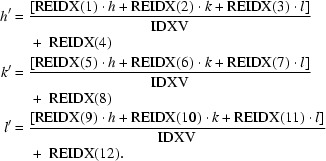
The value of the integer IDXV depends on the lattice type used for specifying reflection indices in the integration step. IDXV is 1 for a primitive lattice, 2 for a face-centred or body-centred lattice, 3 for a rhombohedral lattice and 4 for a lattice centred on all faces. In the example case we have IDXV = 1 because integration was carried out in space group *P*1.

Note also that elements 4, 8 and 12 of the transformation are always 0 in this example. These three extra elements were introduced to provide a simple tool for correcting the indices if all reflections are misindexed by a constant.

### 6.2. Finding possible space groups   

For protein crystals, the absence of parity-changing sym­metry operators restricts the number of possible space groups to 65 instead of 230. Moreover, the determination of correction factors for the integrated intensities does not depend on the presence or absence of any screw axes so that data processing can be finished without this knowledge. This reduces the problem to the identification of an enantio­morphous space group without screw axes that is compatible with the observed lattice symmetry (see above).

For solution of the problem, a quality indicator of the mean variation in the intensities of symmetry-equivalent reflections (*R*_meas_) is calculated for each possible group. The decision for a particular group is then based on Occam’s principle: the selected group must explain all integrated intensities in the data set at acceptable quality, thereby requiring a minimum number of unique reflections.

A suitable redundancy-independent data quality indicator has been suggested by Diederichs & Karplus (1997 ▶) and Weiss (2001 ▶), 
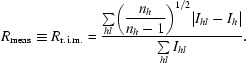
The subscript *h* represents the unique reflection indices and *hl* denotes any of the *n_h_* symmetry-related reflections to *h*. The absolute differences between the observed intensities *I_hl_* and their mean intensity *I_h_* are weighted to remove any dependency on *n_h_* and compared with the intensities. Small values of *R*_meas_ indicate accurate single observations *I_hl_* and the use of symmetry operators compatible with the intensity data set.

For the above example data set, Table 2 ▶ lists all enantiomorphous groups which are in harmony with the observed lattice symmetry shown in Table 1 ▶. For each listed space group, UNIQUE is the number of unique reflections and COM­PARED is the number of reflections used to calculate the redundancy-independent *R* factor *R*_meas_. Two sets of groups can be distinguished clearly: those implying an acceptable *R*_meas_ and a second set with *R*_meas_ > 45%, which is totally unacceptable. Among the acceptable solutions a minimum number of unique reflections is needed if the crystal has the tetragonal space-group symmetry *P*422.

## Figures and Tables

**Table 1:** Rating of lattice types implied by a given reduced cell

			Conventional unit-cell parameters (, )						
Lattice type		Quality index	*a*	*b*	*c*				Reindexing transformation
44	*aP*	0.0	159.3	159.4	160.4	90.1	90.1	90.1	11 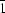 0/1 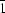 10/ 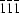 0
31	*aP*	0.4	159.3	159.4	160.4	90.1	89.9	89.9	1000/0100/0010
34	*mP*	1.4	159.3	160.4	159.4	90.1	90.1	90.1	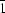 000/00 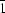 0/0 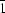 00
14	*mC*	1.4	225.1	225.6	160.4	90.0	90.1	89.9	1100/ 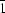 100/0010
33	*mP*	1.5	159.3	159.4	160.4	90.1	90.1	90.1	1000/0100/0010
35	*mP*	2.0	159.4	159.3	160.4	90.1	90.1	90.1	0 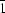 00/ 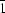 000/00 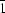 0
13	*oC*	2.3	225.1	225.6	160.4	90.0	90.1	89.9	1100/ 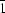 100/0010
32	*oP*	2.4	159.3	159.4	160.4	90.1	90.1	90.1	1000/0100/0010
10	*mC*	2.5	225.1	225.6	160.4	90.0	90.1	90.1	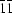 00 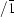 100/00 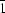 0
**11**	***tP***	**3.4**	159.3	159.4	160.4	90.1	90.1	90.1	1000/0100/0010
25	*mC*	5.9	226.0	226.2	159.3	90.0	90.2	89.7	0110/0 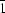 10/1000
20	*mC*	6.4	226.0	226.2	159.3	90.0	90.2	90.3	0 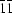 0/0 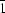 10/ 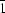 000
4	*hR*	7.4	225.6	226.2	276.2	90.3	89.9	119.9	1 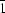 00/ 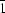 010/ 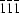 0
23	*oC*	7.8	226.0	226.2	159.3	90.0	90.2	89.7	0110/0 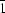 10/1000
3	*cP*	7.8	159.3	159.4	160.4	90.1	90.1	90.1	1000/0100/0010
21	*tP*	8.2	159.4	160.4	159.3	90.1	90.1	90.1	0100/0010/1000
2	*hR*	8.7	225.1	225.8	276.9	90.2	90.0	119.8	1100/ 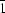 0 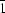 0/ 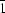 110
5	*cI*	173.6	225.8	225.1	226.0	60.2	59.9	60.2	1010/1100/0110

**Table 2:** Identification of possible space groups

							Conventional unit-cell parameters (, )					
Space group		Lattice type		*R*_meas_ (%)	UNIQUE	COMPARED	*a*	*b*	*c*			
1	*P*1	44	*aP*	5.8	35341	22207	159.3	159.4	160.4	90.1	90.1	90.1
1	*P*1	31	*aP*	5.8	35341	22207	159.3	159.4	160.4	90.1	89.9	89.9
3	*P*2	33	*mP*	6.5	21904	35644	159.3	159.4	160.4	90.0	90.1	90.0
3	*P*2	34	*mP*	7.0	26743	30805	159.3	160.4	159.4	90.0	90.1	90.0
5	*C*2	10	*mC*	7.7	22207	35341	225.1	225.6	160.4	90.0	90.1	90.0
5	*C*2	14	*mC*	7.7	22207	35341	225.1	225.6	160.4	90.0	90.1	90.0
16	*P*222	32	*oP*	7.9	14461	43087	159.3	159.4	160.4	90.0	90.0	90.0
21	*C*222	13	*oC*	8.0	15094	42454	225.1	225.6	160.4	90.0	90.0	90.0
3	*P*2	35	*mP*	8.2	25786	31762	159.4	159.3	160.4	90.0	90.0	90.0
75	*P*4	11	*tP*	8.5	14944	42604	159.4	159.4	160.4	90.0	90.0	90.0
**89**	***P*422**	** 11**	***tP***	**9.0**	8086	49462	159.4	159.4	160.4	90.0	90.0	90.0
146	*R*3	2	*hR*	45.2	20068	37480	225.5	225.5	276.9	90.0	90.0	120.0
5	*C*2	20	*mC*	46.9	23125	34423	226.0	226.2	159.3	90.0	90.2	90.0
5	*C*2	25	*mC*	46.9	23125	34423	226.0	226.2	159.3	90.0	90.2	90.0
75	*P*4	21	*tP*	49.2	14828	42720	159.9	159.9	159.3	90.0	90.0	90.0
89	*P*422	21	*tP*	50.7	7876	49672	159.9	159.9	159.3	90.0	90.0	90.0
21	*C*222	23	*oC*	51.3	15155	42393	226.0	226.2	159.3	90.0	90.0	90.0
195	*P*23	3	*cP*	57.3	5344	52204	159.7	159.7	159.7	90.0	90.0	90.0
207	*P*432	3	*cP*	58.1	2896	54652	159.7	159.7	159.7	90.0	90.0	90.0
155	*R*32	4	*hR*	59.7	9038	48510	225.9	225.9	276.2	90.0	90.0	120.0
155	*R*32	2	*hR*	60.7	10487	47061	225.5	225.5	276.9	90.0	90.0	120.0
146	*R*3	4	*hR*	61.1	16751	40797	225.9	225.9	276.2	90.0	90.0	120.0
